# The role of p53 in liver fibrosis

**DOI:** 10.3389/fphar.2022.1057829

**Published:** 2022-10-24

**Authors:** Siyu Yu, Guang Ji, Li Zhang

**Affiliations:** Institute of Digestive Diseases, Longhua Hospital, Shanghai University of Traditional Chinese Medicine, Shanghai, China

**Keywords:** liver fibrosis, p53, hepatic stellate cells, apoptosis, ferroptosis

## Abstract

The tumor suppressor p53 is the central hub of a molecular network, which controls cell proliferation and death, and also plays an important role in the occurrence and development of liver fibrosis. The abundant post-translational processing and modification endow the functional diversity of p53. Considering the relationship between p53 and liver fibrosis, drug intervention targeting p53 or management of p53 regulation might be effective strategies to treat liver fibrosis. Here, we systematically discuss the regulation of p53 in different liver cells (hepatocytes, immune cells, HSCs, *etc*) and the role of p53 in the development of liver fibrosis, and propose possible interventions to prevent the pathogenic processes of liver fibrosis.

## Introduction

Liver fibrosis is a complex fibrogenic and inflammatory process, characterized by the accumulation of extracellular matrix (ECM), which distorts the hepatic architecture, and potentiates risks for cirrhosis and even hepatocellular carcinoma. Liver fibrosis generally results from chronic liver-damaging factors such as virus, alcohol, nonalcoholic steatohepatitis (NASH), cholestasis, autoimmune diseases (e.g., primary biliary cirrhosis, primary sclerosing cholangitis), drugs, parasitic infections, and genetic mutations (Acharya et al., 2021). All these hepatocellular injuries can activate the fibrogenic pathways and hepatic stellate cells (HSCs), and promote the differentiation of ECM-producing myofibroblasts (MFBs). HSCs are considered the main effector cells of liver fibrosis, quiescent HSCs reside in the Disse space and are the lipid storage cells, whereas the activation of HSCs involves a host of phenotypic changes, including loss of lipid droplets, trans-differentiation into MFBs, and expression of contractile fibers.

Physiologically, ECM is part of the boundary between blood flow and parenchyma, and fibrogenesis is a normal wound healing response. During the reparative process, ECM is made up of glycoproteins, proteoglycans, and non-fibrogenic type IV collagen, these components form a lattice-like matrix to support the proper arrangement and function of liver cells. However, upon various injuries, the synthesis of ECM is accelerated, and the non-fibrogenic type IV collagen is replaced by fibrogenic type I and II collagen, and the change of composition and density also alters the structure of the matrix and disrupt the normal structure of the liver (Bataller and Brenner, 2001). If the injury is acute or self-limiting, fibrogenic changes are transient and reversible. Once the primary disease is controlled or tissue damage is reduced, the activated HSCs can return to a quiescent state (Hernandez-Gea and Friedman, 2011; Seki and Brenner, 2015). Actually, spontaneous regression of liver fibrosis can be observed on day 28 after carbon tetrachloride (CCl_4_) intraperitoneally injections (Iredale et al., 1998). However, if the inflammatory response and the injury persist, the liver parenchyma is gradually replaced by scar tissues, which may further develop into cirrhosis and liver failure (Aydin and Akcali, 2018).

Currently, specific antifibrotic agents to treat liver fibrosis are still not available albeit several novel agents have entered the pre-clinical stage. Presently, clinical practice guidelines for liver fibrosis are only etiology-specific, to prevent progression and target the cause of liver injuries, such as antiviral drugs, alcohol withdrawal, treatment of metabolic disorders, and weight loss (Yamada et al., 2018; Damiris et al., 2020). The pathogenesis of liver fibrosis is rather complex, which involves cell-cell communication (e.g., hepatocytes and HSCs, liver macrophages and HSCs), activation and modulation of different signaling pathways, and immune system and tissue repair pathways among others. Therefore, understanding the mechanisms is fundamental for developing practicable drugs for liver fibrosis.

Since advanced liver fibrosis has the potential to progress into hepatocellular carcinoma, liver fibrosis is also considered a precancerous pathology. P53 is a well-known tumor suppressor gene, locates on human chromosome 17 with a relative molecular mass of about 53 kDa. Recent studies revealed that p53 also plays an important role in the development and progression of fibrosis. P53 interacts with fibrogenesis and fibrinolysis pathways, and mice with p53 deficiency spontaneously develop into liver fibrosis, suggesting that p53 is an important regulator of liver fibrosis.

## P53 is required for the anti-fibrosis process

**FIGURE 1:**
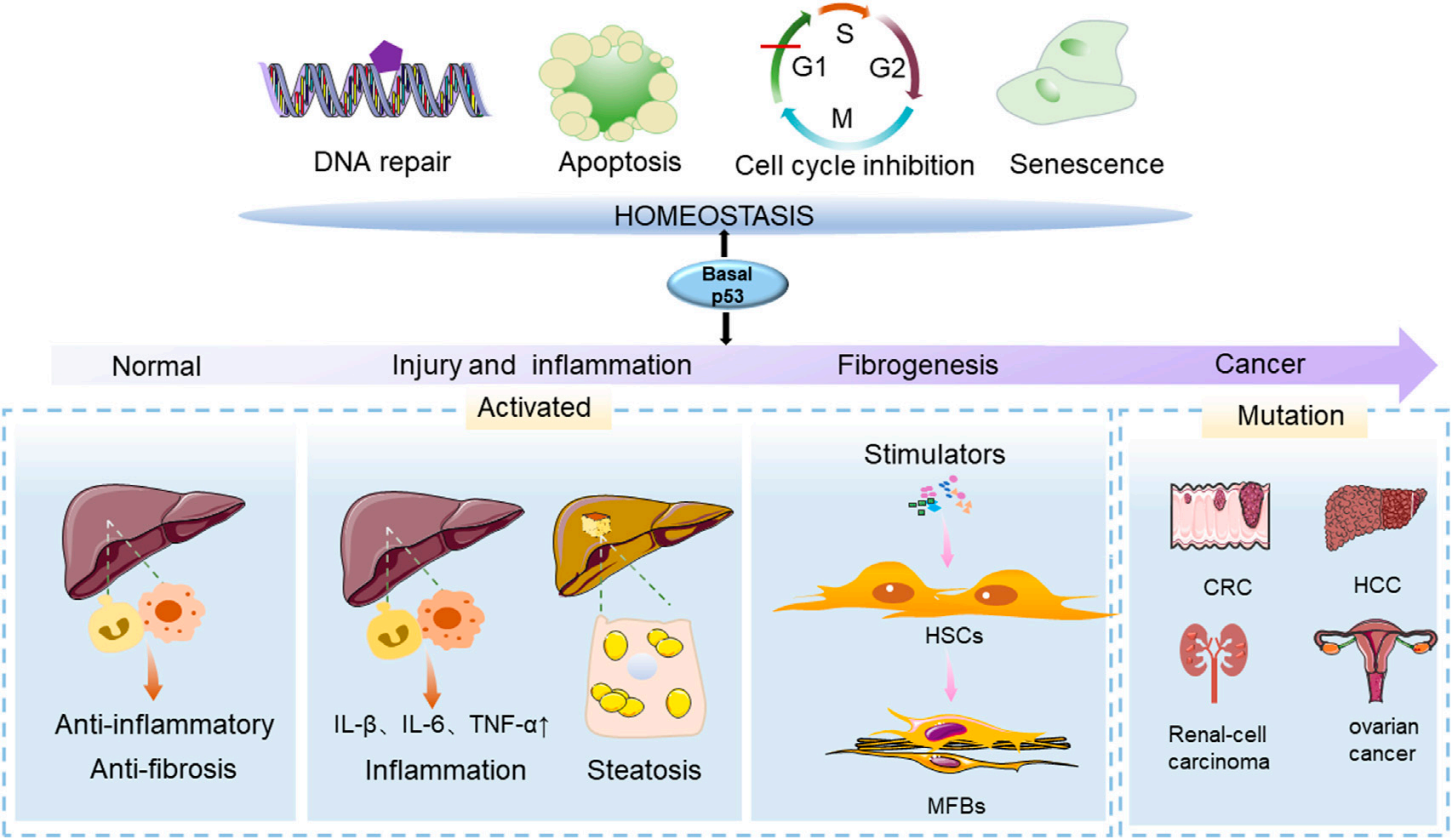
The function of p53 in physiological and pathological conditions of the liver. The p53 gene (TP53) is an important regulator in maintaining cellular homeostasis and can control cellular survival processes such as cell cycle arrest, apoptosis, senescence, DNA damage and differentiation. The effects of p53 on the liver are varied at different stages of liver injury. During the initial stage of p53 activation, it has anti-inflammatory and anti-fibrotic properties. In the presence of chronic liver injury, p53 activation and senescence in HSCs promote HSC accumulation, leading to the development of liver disease. Mutated p53 acquires carcinogenic properties, and contributes to the development of cancers.

P53 gene (TP53) is crucial for maintaining genome integrity and intracellular homeostasis *via* initiating the survival process of cells such as cell cycle arrest, apoptosis, senescence, DNA damage and differentiation (Kanapathipillai, 2018; Lv et al., 2018). P53 protein locates on human chromosome 17 with a relative molecular mass of about 53 kDa. Physiologically, p53 protein is unstable and maintained at low levels under the action of negative regulatory factors in cells (Brooks and Gu, 2006; Blandino and Di Agostino, 2018) (Figure 1). However, TP53 is extremely susceptible to mutating all cancer types (Zhu et al., 2015; Sabapathy and Lane, 2019), it undergoes a variety of mutations such as point mutation, deletion, frameshift and rearrangement (Wu et al., 2020). When TP53 is mutated, the encoded P53 protein obtains a prolonged half-life and strong stability, which continuously accumulates in the nucleus and loses its monitoring functions (Itahana and Itahana, 2018). Mutated p53 acquires carcinogenic properties, including cell proliferation, chemoresistance, disruption of tissue structures, promotion of migration, invasion and metastasis (Blandino and Di Agostino, 2018).

The transforming growth factor beta (TGF-β) pathway determines the synthesis and degradation of ECM during the process of fibrosis (Muthuramalingam et al., 2020). TGF-β binds to its receptors and recruits smooth muscle actin (Smad) and P53 to form transcriptionally-active multi-protein complexes. It is reported that increased p53^S15^ phosphorylation accelerates renal damage and compromised organ function in experimental renal injury animals (Muthuramalingam et al., 2020). Tubular-specific p53 ablation or p53 inactivation in mice prevents epithelial G2/M arrest, reduces the secretion of fibrotic effectors, and attenuates the transition from acute to chronic kidney injury (Yang et al., 2010). In the presence of hypoxia, HIF-1α up-regulates p53 and activates TGF-β and CTGF-mediated signaling pathways, leading to ECM formation and renal fibrosis (Liu et al., 2019). Active TGF-β1 and p53 signaling pathways can be also observed during myocardial fibrosis in the heart tissue of dilated cardiomyopathy patients with ventricular tachycardia (Haywood et al., 2020). The use of Bleomycin can aggravate the process of pulmonary fibrosis by upregulating the expression of P53 and P21 in A549 human alveolar epithelial cells (Muthuramalingam et al., 2020). In response to silica, upregulation of p53 activates the RMRP/miR122 signaling pathway and promotes epithelial-mesenchymal transition (EMT), leading to the progression of pulmonary fibrosis in BALB/c mice (Li et al., 2021). Accordingly, the lung tissue damage and collagen deposition in p53 deficient mice are significantly reduced compared with wild-type mice, suggesting that inhibition of p53 expression could delay the progression of lung fibrosis (Wu et al., 2020).

The development of liver fibrosis attributes to HSC activation as well as the pathological change of hepatocytes and liver macrophages among others. Growing research suggests p53 accumulation in hepatocytes of several fibrotic liver diseases, such as NASH, viral hepatitis and primary biliary cirrhosis. The expression of the pro-apoptotic protein p53 is increased while anti-apoptotic protein Bcl-2 is inhibited with the enhancement of the inflammatory response in non-alcoholic fatty liver disease (NAFLD) patients (Panasiuk et al., 2006). Overexpression of p53 can be observed in 35% of samples in liver biopsies in patients with non-neoplastic liver disease, steatohepatitis and chronic hepatitis (Akyol et al., 1999). A study of p53 expression in the liver of patients with HCV infection showed that p53 was overexpressed in 7 of 40 patients (17.5%), indicating that overexpression of p53 may occur in the early stages of HCV-related liver disease (Rektorova et al., 2003). Liver macrophages includes the resident Kupffer cells and recruited liver macrophages. The crosstalk of hepatocytes, macrophages and HSCs can be triggered and facilitated by a range of chemical mediators, of which transforming growth factor beta (TGF-β) plays a prominent role (Poli, 2000).

Mutant p53^R172H^ is associated with spontaneous liver inflammation and steatosis when combined with the loss of IL27 signaling (IL27RA), and mice develop microscopic and macroscopic steatosis, hepatocyte necrosis, immune cell infiltration and fibrosis with age (Dibra et al., 2016). P53 contributes to fibrotic disease progression by downregulating sirtuins in the liver, leading to telomere dysfunction. By inhibiting p53, mitochondrial function and liver fibrosis can be improved to some extent (Amano et al., 2019). IL-10 gene therapy can alleviate liver fibrosis in rats upon CCL_4_ injection by restoring HSC senescence in the fibrotic liver, however, the effect is abrogated in p53 knockout rats, indicating p53 is required for the anti-fibrosis effect of IL-10 (Guo et al., 2021). Collectively, these studies suggested that the increase of p53 in response to various liver stimulation might a stress reaction to prevent disease progression, and p53 is involved and required in the anti-fibrosis process.

## P53 interacts with fibrosis signaling

The stressed environment of the liver often leads to the activation of p53, resulting in changes in metabolic pathways and inducing apoptosis (Charni et al., 2014). Mdm2 is a protein that promotes p53 degradation, hepatocyte-specific Mdm2 knockout mice present endogenous p53 protein accumulation, which further upregulates connective tissue growth factor (CTGF) and formation of spontaneous liver fibrosis (Kodama et al., 2011). P14 Cdk1Liv^−/−^ mice mimic the lack of division ability of hepatocytes in chronic hepatitis C (CHC) patients, and enhanced p53 signaling can be observed in the liver, accompanied by the progression to liver inflammation and fibrosis (Dewhurst et al., 2020). In methionine-and choline-deficient diet-fed mice, serum IGF-1 levels decreased with the progression of simple steatosis to NASH, and the expression of p53 and its downstream target gene p21 in the liver also increased, which might be involved in initiating cell apoptosis and enhance clearance of damaged hepatocytes (Farrell et al., 2009). P53 can directly regulate the expression of specific microRNAs, the most significant of which is the miR-34 site, including miR-34a, miR-34b, and miR-34c (Vousden and Prives, 2009). MiR-34a/SIRT1/p53 signaling pathway is activated in hepatocytes of CCL_4_-induced fibrotic rats, leading to hepatocyte apoptosis, thereby activating hematopoietic stem cells and participating in the process of liver fibrosis (Tian et al., 2016). Levels of collagen markers in serum and the expression of p53 in liver tissue are positively correlated with serum miR-34a in CHC patients (Li et al., 2020). Triclosan induces liver injury in zebrafish by triggering the abnormal expression of miR-125 that is mediated by the MAPK/p53 signaling pathway (Guo et al., 2021).

Monocytes and macrophages are known sources of TGF-β, and upon chronic liver injury, the secretion of TGF-β is significantly increased, which has a specific stimulatory effect on collagen formation. Experiments demonstrate that Kupffer cells isolated from alcoholic fibrosis rat liver express and release TGF-β, and Smad and p53 protein complexes synergistically activate TGF-β-induced transcription. P53 promotes the activation of multiple TGF-β target genes during embryonic development in *Xenopus*. In mammalian cells, TGF-β requires p53 for complete transcriptional activation of CDK inhibitor p21WAF1, and p53 deficient cells show impaired cellular inhibitory responses to TGF-β signaling (Cordenonsi et al., 2003). Loss of type II TGF-β receptor inactivated TGF-β signaling synergizes with inactivated p53 to promote hepatocellular carcinoma (Morris et al., 2012).

Hepatocyte apoptosis induced by p53 may lead to inflammatory cell infiltration, liver cirrhosis, and even liver cancer in the long term (Charni-Natan et al., 2019). In primary hepatocytes, TGF-β treatment increased the p53 and p66Shc signaling pathways, leading to excessive accumulation of reactive oxygen species (ROS) and apoptosis. The liver p53 and p66Shc signaling pathways are enhanced in a mouse model of NASH, and p53 deletion can inhibit the enhanced p66Shc signaling, reduce hepatic lipid peroxidation and the number of apoptotic hepatocytes, and improve the progression of nutritional steatohepatitis. The expression levels of p53, p21, and p66Shc are significantly elevated in liver specimens from NAFLD patients (Tomita et al., 2012). In primary rat hepatocytes, TGF-β1 trans-activates E2F-1, leads to Mdm-2 degradation and increases the expression of p53, and the levels of BAX protein and mRNA are significantly increased to induce hepatocyte apoptosis (Sola et al., 2003). Activation of p53 and TGF-β1/Smads signaling pathways leads to 10% fructose-induced epithelial-mesenchymal transition in rat hepatocytes, leading to liver fibrosis (Song et al., 2019).

## P53 mediates the function of HSC

**FIGURE 2:**
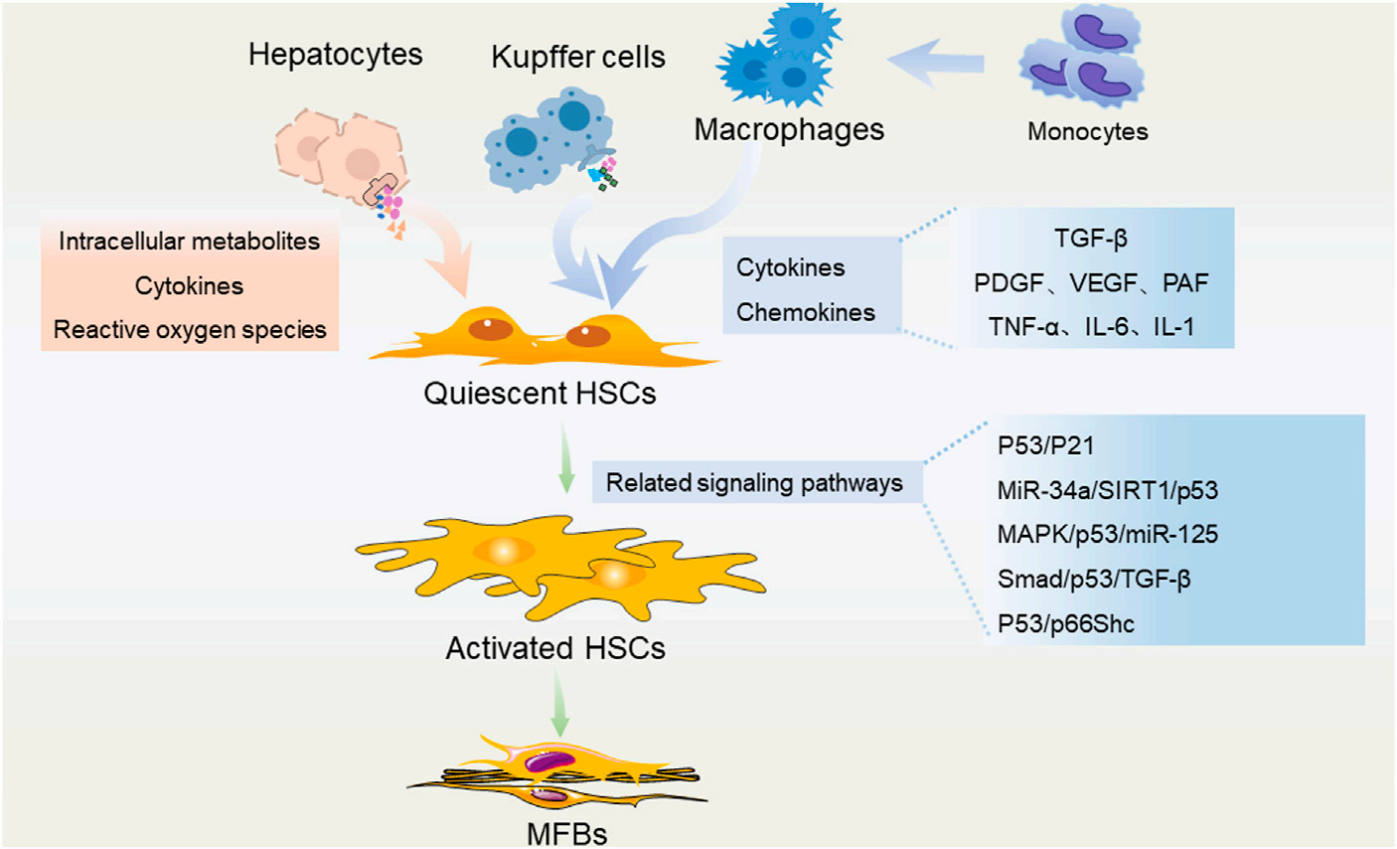
P53 in regulating different liver cells during the development of liver fibrosis. Hepatocytes, liver macrophages (Kupffer cells and recruited monocyte-derived macrophages) secrete a variety of intracellular metabolites, cytokines, chemokines, reactive oxygen species, etc., and activate HSCs through a variety of p53-related pathways. These pathological stimulations could promote the trans-differentiation of HSCs into MFBs, and subsequently lead to liver fibrosis.

After an acute injury, activated HSCs can support hepatocyte proliferation and tissue repair, and as injury continues, activated HSCs migrate and accumulate at tissue repair sites, tans-differentiated into MFBs to secrete large quantities of ECM and initiate the liver fibrosis process (Bataller and Brenner, 2005; Passino et al., 2007). Therefore, inhibiting HSC activation and inducing the cell death of HSC are equally important in the anti-fibrosis process (Figure 2). Hepatocyte death and inflammation are reduced after neddylation inhibition, which may partially explain the reduction in HSC activation (Author Anonymous., 1989). As aforementioned, p53 is an active regulator of TGF-β1 secretion from hepatocytes and immune cells, and the quantity and stability of p53 in HSCs are necessary to block the development of fibrosis.

Promoting apoptosis is a promising strategy for fibrosis (Xue et al., 2022). As a well-known cancer suppressor, p53 is actively involved in cell apoptosis, ferroptosis, and senescence of cells. Activation of the p53/Bax/Bcl-2 signaling pathway promotes cell apoptosis, inhibits cell proliferation and migration, and reduces the production of ECM. In addition, increased p53 is associated with ROS production, which might further enhance cell apoptosis. P53 also ferroptosis to eliminate damaged cells (Wu and Prives, 2018; Stein et al., 2019). BRD7 directly binds to the N-terminal transactivation domain to promote mitochondrial translocation of p53, which in turn forms a complex with SLC25A28 to enhance the activity of SLC25A28, resulting in abnormal accumulation of redox-active iron and electron transfer chain hyperfunction, enhanced ferroptosis in HSCs and improved the degree of liver fibrosis in mice (Zhang et al., 2020). P53 is an upstream molecule that promotes artemether-induced ferroptosis in HSCs, and inhibits HSC activation accordingly. In CCL_4_-induced liver fibrosis, artemether significantly downregulated many markers of HSC activation, including α-SMA, Col1a1, and fibronectin, and inhibited profibrotic receptors such as TGF-βR1, PDGF-βR and epidermal growth factor receptor (Wang et al., 2019).

Senescent cells also contribute to the generation of ECM and fibrotic scars during liver injury (Kim et al., 2013). In mice lacking key regulators of aging, HSCs obtain a sustaining proliferation and cause subsequent liver fibrosis. Transcriptomic analysis of senescent and apoptotic cells revealed that LY6D expression is enhanced in senescent cells in a p53-dependent pattern (Hu et al., 2022). P53 promotes senescence of activated HSCs during acute liver injury, simultaneously, decreased ECM and downregulated ECM-degrading enzymes can be observed. Natural killer (NK) cells are reported to preferentially clear senescent HSCs *in vitro* and *in vivo*, thereby protecting the liver from excessive fibrotic responses (Krizhanovsky et al., 2008). P53 can restrict malignant transformation by triggering cell-autonomous programs of cell-cycle arrest and cellular senescence. It is reported that ablation of a p53-dependent senescence program in HSCs increases liver fibrosis and cirrhosis, and enhances the progression to hepatocellular carcinoma (Lujambio et al., 2013).

## Management of p53 is a promising strategy for liver fibrosis

Currently, eliminating irritation or ameliorating the cause of chronic liver diseases, such as the use of antiviral drugs, alcohol withdrawal, treatment of fatty liver disease and weight loss, are the common strategies for preventing liver fibrosis (Trautwein et al., 2015). However, in cases of advanced fibrosis, liver transplantation remains the only effective option (Taymouri and Taheri, 2016). As p53 is required for the anti-fibrosis process, management of p53 is a promising strategy.

Post-translational processing and modification determines the stability and transcriptional activity of p53 protein, and contribute to functional diversification. Phosphorylation is the most common post-translational modification of p53, which variously occurs at the N-terminus, DNA binding domain, C-terminus of p53, etc. Phosphorylation enhances the stabilization and transcriptional activity of p53 (Nguyen et al., 2014; Lv et al., 2018). Ubiquitination of p53 is mediated by E3 ubiquitin ligase (Cubillos-Rojas et al., 2017), ubiquitination and degradation of p53 disrupt p53-dependent transcription, and affect p53-promoted cell growth inhibition, G1 block and apoptosis (Sun et al., 2009; Sun et al., 2011). Multiple lysines at the carboxy terminus are major targets for the regulation of p53 acetylation (Gu and Roeder, 1997; Vaziri et al., 2001). Acetylation of p53 can increase the stability of p53 and play an important role in the activation of downstream target genes. Acetylated p53 (Ac-p53) improves the ability of p53 to bind to DNA, regulates the separation and distribution of p53 between the cytoplasm and nucleus, and promotes the recruitment of coactivators (Oda et al., 2000; Hofmann et al., 2002).

P53 can inhibit cell proliferation or induce apoptosis in tumor cells, as a key inhibitor of p53, Mdm2 has a high affinity to p53 protein. Overexpression of Mdm2 effectively inhibits the function of p53, and Mdm2 and its homolog Mdm4 are commonly overexpressed in human tumors (Grier et al., 2006). The use of potent and selective small molecule Mdm2 antagonists can disrupt the p53-Mdm2 interaction and activate the p53 pathway in cancer cells, resulting in cell cycle arrest, apoptosis, and inhibition of human tumor growth in nude mice (Vassilev et al., 2004). In addition to Mdm2, some ubiquitin ligases such as PIRH2 and COP1 can also promote the degradation of p53 (Collavin et al., 2010). Pifithrin -α, an inhibitor of p53, can reduce the level of nuclear p53 and reduce the activity of caspase3, thus alleviating apoptosis and necrosis (Schafer et al., 2003). Ursodeoxycholic acid (UDCA) specifically inhibits the E2F-1/p53 apoptosis pathway, reduces the stability of p53, decreases NF-κB degradation and downregulates Bcl-2, and alleviated TGF-β1-induced hepatocyte apoptosis in rats (Sola et al., 2003).

## Conclusion and perspectives

Here we reviewed the pathogenesis of liver fibrosis and the research progress of tumor suppressor p53 in liver fibrosis. During fibrosis, the interaction between parenchymal cells and non-parenchymal cells, the activation of different immune cells and signaling pathways, and the release of various inflammatory mediators lead to the occurrence of inflammation, then activate HSCs, lead to the accumulation of ECM, and fibrosis scarring. P53 plays an inhibitory role in various tumor diseases, and mutated or modified p53 is endowed with different functions. In addition to its important role in the occurrence and development of tumors, p53 is involved in fibrosis of different organs such as the liver, kidney, lung and heart, and management of p53 is found to be beneficial for all kinds of fibrosis.

Despite significant progress in basic research on liver fibrosis, the anti-fibrotic activity of many compounds has been demonstrated *in vitro* and in animal models (Trautwein et al., 2015), however, sensitive and specific biomarkers as non-invasive diagnostic tools and effective anti-fibrosis drugs have not yet been developed. The tumor suppressor 53 can regulate the fibrogenic process and potentiate a promising perspective for liver fibrosis. Each member of the P53 family has slightly different roles in tumors, so whether they also play different roles in the development of liver fibrosis is also worthy of further study. P53 regulates lipid metabolism, inflammation, and adipose tissue metabolism, indicating that metabolic-associated liver fibrosis might be more specific for p53 regulation. Therefore, a comprehensive understanding of the relationship between p53 and liver fibrosis is of great significance for the development of target drugs for the treatment of liver fibrosis.
